# Rheological Issues in Carbon-Based Inks for Additive Manufacturing

**DOI:** 10.3390/mi10020099

**Published:** 2019-01-29

**Authors:** Charlie O’ Mahony, Ehtsham Ul Haq, Christophe Silien, Syed A. M. Tofail

**Affiliations:** Department of Physics, and Bernal Institute, University of Limerick, National Technological Park, Limerick V94 T9PX, Ireland; Ehtsham.U.Haq@ul.ie (E.U.H.); Christophe.Silien@ul.ie (C.S.)

**Keywords:** carbon Inks, rheology, additive manufacturing, graphene, carbon nanotubes, printing

## Abstract

As the industry and commercial market move towards the optimization of printing and additive manufacturing, it becomes important to understand how to obtain the most from the materials while maintaining the ability to print complex geometries effectively. Combining such a manufacturing method with advanced carbon materials, such as Graphene, Carbon Nanotubes, and Carbon fibers, with their mechanical and conductive properties, delivers a cutting-edge combination of low-cost conductive products. Through the process of printing the effectiveness of these properties decreases. Thorough optimization is required to determine the idealized ink functional and flow properties to ensure maximum printability and functionalities offered by carbon nanoforms. The optimization of these properties then is limited by the printability. By determining the physical properties of printability and flow properties of the inks, calculated compromises can be made for the ink design. In this review we have discussed the connection between the rheology of carbon-based inks and the methodologies for maintaining the maximum pristine carbon material properties.

## 1. Introduction

**Figure 1:**
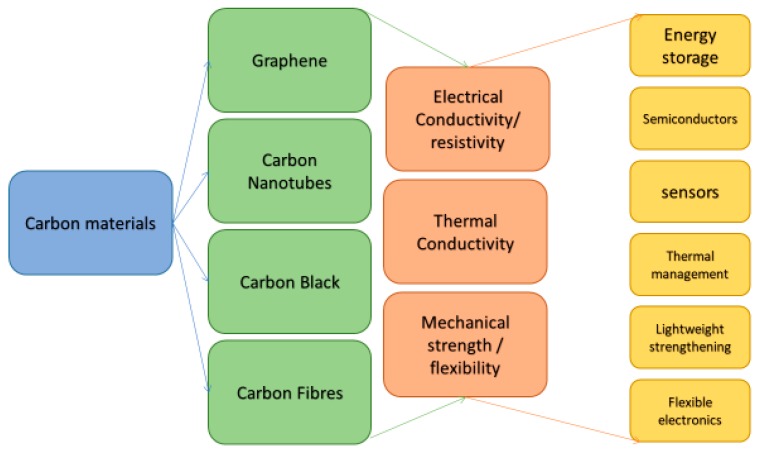
Outline of various forms of carbon (C) used for inks in additive manufacturing and conventional printing techniques, along with their beneficial properties and potential uses.

Carbon (C) is a many allotrope material that can exist in various forms. These forms vary from diamond, an ultra-hard optically isotropic (directionally transparent or opaque) to graphite, a soft grey material [1]. This high variability in properties gives rise to a similarly high amount of utility. Carbon allotropes such as Graphene and Carbon nanotubes pose interesting properties in surface areas, tensile strength, low density, stretchability, thermal conductivity, current density, gas impermeability, and overall electrical properties [2,3,4,5,6,7]. An outline of the important properties and potential uses is seen in Figure 1.

Conventionally, carbon is solid in nature, arising from strong covalent bonds, which makes printing it alone impossible, with exceptions at extreme temperature and pressure. To utilize these materials in printing methods, the particles must be suspended in a fluid and used as a vehicle for printing. This mixture of solid and liquid forms is known as a colloidal system. 

Colloidal systems are heterogeneous solutions with a dispersed phase uniformly distributed throughout the second dispersion phase. This system presents interesting rheological properties, which bears importance due to the use of these colloidal inks in additive manufacturing and conventional 2D printing processes. Printability determines the finished print quality in terms of porosity, surface finish, and resolution of geometry. The determination of ideal printability requires tailored rheological properties while maintaining the intended properties of the novel carbon particles [4,5,6,7,8,9,10,11,12].

**Figure 2:**
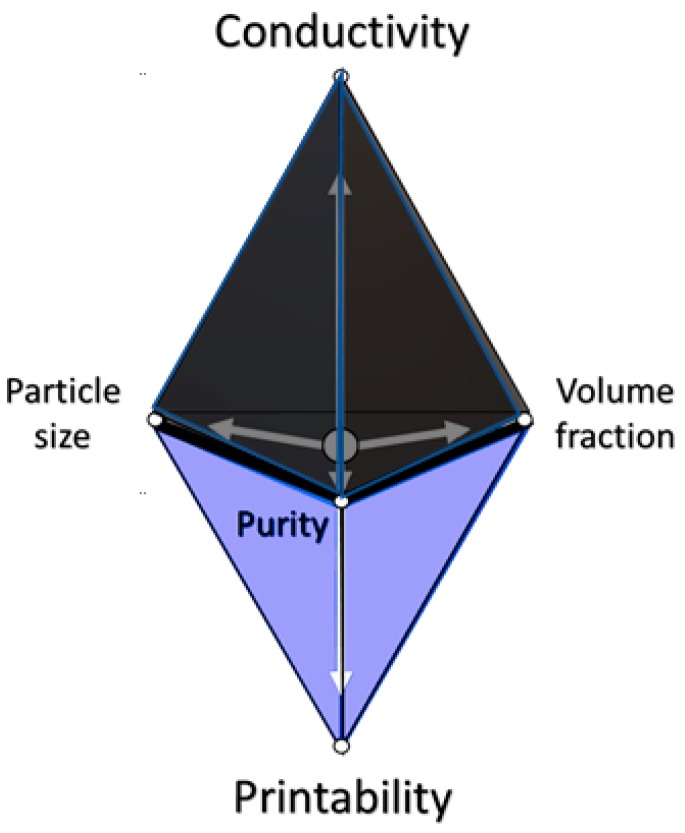
Graphical representation of the general relationship of the conductivity trade-off with printability, in terms of tailoring conductive inks. The variables in the base triangle increase (particle size (μm), volume fraction (%), and purity (%)) the conductivity increases. Conversely, the printability increases with decreases in these variables.

Rheology is the study of the deformation and flow of matter, which holds a great amount of relevance to ink printing. Designing the flow of inks is of great importance, as the ease at which the ink flows and stiffens when shaped into the intentional design is key. Efforts to determine the idealized flow properties must be investigated to ensure maximum printability. A representation of this compromise of properties for printability is seen in Figure 2. Since these carbon-based inks are colloidal, a clear understanding of the flow nature of the suspended particles in additive the manufacturing and conventional printing methods will dictate the overall printability of the carbon allotropes.

This review paper intends to bridge the gap between the rheological importances to printing inks while maintaining the intended properties of the printed product. It is clear that there is no one size fits all when weighing the rheological properties versus the desired properties. One of these properties is conductivity, as both graphene and carbon nanotubes present usability in semiconductors and electronic circuits, as examples. A large conductivity range opens the inks to a plethora of purposes, which require important controls. Previous works investigating this have been done by Lucja Dybowska-Sarapuk et al. [6], where the various carbon-based inks were compared rheologically. M.I. Maksud et al. [7] investigated the printability of carbon nanotubes, though understanding how to optimize the printability of idealized material properties in carbon-based inks is still absent in the literature. A review paper of relevance was conducted by Derby [8], in which the rheological aspects of 3D printing ceramics were investigated.

## 2. Background

The process capabilities of printing go hand in hand with the material’s rheological and mechanical properties. Printing performance, or the “printability” of ink, is defined by many physical parameters and fluidic properties, such as density (*ρ*), viscosity (*η*) and surface tension (*σ*). When considering the carbon-based inks used in 3D printing, these properties must be accounted for to ensure high printability and resolution [9].

### 2.1. Rheology

Rheology is the study of flow and the structural properties of viscous materials. To investigate these flow properties, rotational shear is applied, causing the sample to flow. There are two methods to investigate flow properties: Linear (oscillatory) and Non-linear (Steady-Shear) [10].

#### 2.1.1. Linear Rheology

Small amplitude oscillatory shear (linear rheology) is widely used in viscoelastic material characterization. It is a non-destructive method. Due to the small amplitude the deformation does not exceed the linear viscoelastic region of the material. This is achieved through cyclically varying stress and strain in a sinusoidal fashion. The material responds elastically to deformation, rather than plastic deformation within this region. Contributions from the viscous and elastic responses in the material are measured, this gives both complex modulus G* (comprised of loss and storage) and the phase angle *δ*. During this test, the sample is under harmonic strain causing harmonic stress. The harmonic strain can be represented by:

(1) $$\gamma =\gamma_{0}\sin\left(\omega t\right)$$

where *γ* is the strain, *γ*_0_ is the strain amplitude, ω is the angular frequency of oscillation and *t* is the time. The stress varies with the same angular frequency, *ω,* amplitude of *σ*_0_. However, it is out of phase with the strain by an angle, *δ*. The linear response of the material in terms of stress can be written as:

(2) $$\sigma =\sigma_{0}\sin\left(\omega t+\delta \right)$$

This equation is only valid at low strain amplitudes. At larger strains, however, a non-linear response is observed in the sample. The strength of small amplitude shear oscillation is that the stress response gives quantifiable material values, in storage (*G’*) and loss (*G’’*) moduli. These moduli are the ratios of stress and strain amplitudes, storage being the real (elastic response, in-phase stress) and loss being the imaginary (viscous response, out-of-phase stress). Equation (2) can be further described as [12]

(3) $$\sigma =G^{\prime }\left(\omega \right)\sin\omega t+G\prime \prime \left(\omega \right)\cos\omega t$$

Maxwell created a model based on the linear region of viscoelastic behavior, comparing it to that of a spring and a dashpot in series, the total strain consisting of the spring strain (*ε*_1_) and the dashpot strain (*ε*_2_). With the strain the same in both elements, we are given the equations:

(4) $$\begin{array}{ccc} \varepsilon_{1}=\frac{1}{E}\sigma & \dot{\varepsilon_{2}}=\frac{1}{\eta }\sigma & \varepsilon =\varepsilon_{1}+\varepsilon_{2} \end{array}$$

Differentiating the left and right equations above with respect to time and inputting the left and middle into the right we get:

(5) $$\dot{\varepsilon }=\frac{1}{E}\dot{\sigma }+\frac{1}{\eta }\sigma$$

We put this in the standard form with the stress on the left and strain on the right, giving Maxwell’s equation:

(6) $$\sigma +\frac{\eta }{E}\dot{\sigma }=\eta \dot{\varepsilon }$$

*η* is the viscosity, *E* is Young’s modulus [12].

#### 2.1.2. Non-Linear Rheology

Steady-state shear experiments consist of a continuous stress sweep. Because the large deformities created during the test promote deviation for the linear relationship between the shear stress and shear rate. These deformities make this method non-linear and destructive towards the sample. This test is commonly applied to complex interfaces like emulsions, foams, biological fluids, polymers and colloidal particles, and dispersion of vesicles etc., which tend to have a non-linear response to applied deformations even if they are relatively small. This form of experimentation gives the fluid type (Newtonian, shear thinning, etc.). The properties related to this are:

$$Shear\;rate=\frac{d\gamma }{dt},\;Shear\;stress,\;\sigma ,\;viscosity,\;\eta$$

The viscosity is the proportionality constant between the shear rate and the shear stress.

### 2.2. Rheological Connection to Additive Manufacturing

In this review, the importance of these rheological properties will be discussed via their impact in 3D printing techniques. A factor of practicality to 3D printing is the non-Newtonian fluid type called “shear-thinning”. Shear-thinning is a phenomenon in which the viscosity of the fluid decreases with increasing shear stress. Shear thinning can be time-dependent, this behavior is called thixotropic. This is characterized by the fluidification of the material under shear stress and stiffening at rest. It is a reversible property of the material [13]. Thixotropic materials show shear rate dependent rheological properties. Benefits arise from this property in inkjet printing, as the fluid has high viscosity under standard conditions but low viscosity when passing through the print head. This avoids clogging and fluidizes through the nozzle. Once the drop is detached from the nozzle, the viscosity increases again, suppressing satellite drop formations. Solidification, when deposited, is also improved [14]. 

Shear thinning suppresses satellite droplets, as shown by Hoath et al. and confirmed with work from Morrison and Harlen [8,9]. Satellite drops are partial, unintended droplets between the drop stream formed from surface tension. These shear thinning effects on satellite droplets formation have been studied by Hoath et al. in aqueous PEDOT:PSS. These satellites reduce printability performance, in terms of resolution [15].

### 2.3. Factors Affecting Rheology

#### 2.3.1. Temperature

Viscosity is highly sensitive to temperature, making it a very important factor in processing conditions and end result quality. An increase in temperature creates thermal motion of the molecules, resulting in a displacement that overcomes the intermolecular interactions. With increasing temperature, viscosity decreases, as predicted by the Arrhenius equation:

(7) $$\eta =\eta_{\infty }e^{\frac{Ea}{RT}}$$

where *η* is the viscosity, $\eta_{\infty }$ is the pre-exponential factor, Ea is the activation energy for flow, R is the universal gas constant, and *T* is the absolute temperature in Kelvin and the Boltzmann constant R [9,10]. This equation assumes that there are no physical/chemical changes being induced by the applied heat energy. 

#### 2.3.2. Pressure

Viscosity is largely dependent on the free volume of the system. Since the free volume of a system is influenced by pressure, the viscosity is pressure dependent. The pressure reduces free volume and as a result, it reduces molecular mobility. This, however, becomes noticeable only at high pressures. The rise in pressure increases both the *T*_g_ and *T*_m_, which also reflects an increase of viscosity [16]. Viscosity generally increases with increasing pressure; the general correlation is given by:

(8) $$\eta =A_{0}^{\prime }e^{B_{0}^{\prime }p}$$

where $A_{0}^{\prime }$ and $B_{0}^{\prime }$ are constant and *p* is the pressure.

#### 2.3.3. pH

**Figure 3:**
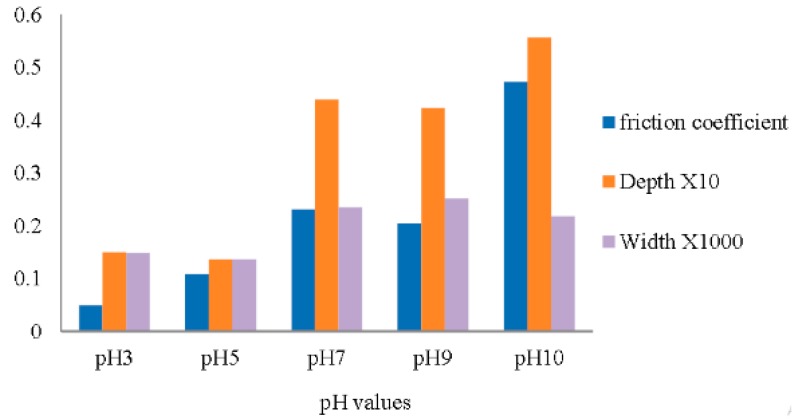
Dependence of the flow behavior on pH level of graphene oxide (GO) in water lubrication, from this graph we observe the trend of higher friction being created. Taken from Aias et al. open access © University Malaysia Pahang Publishing, Malaysia [17].

From one investigation by Alias et al., the dependence of pH on rheology was observed to be that friction increases with increasing pH. Highly acidic graphene suspensions showed lower friction/viscosity. This is associated with higher pH increasing agglomeration and reduced dispersions. As seen in Figure 3. In low pH levels, graphene oxide (GO) sees better dispersion in distilled water, reducing the friction coefficient. 

#### 2.3.4. Topography & Shape of the Suspension

**Figure 4:**
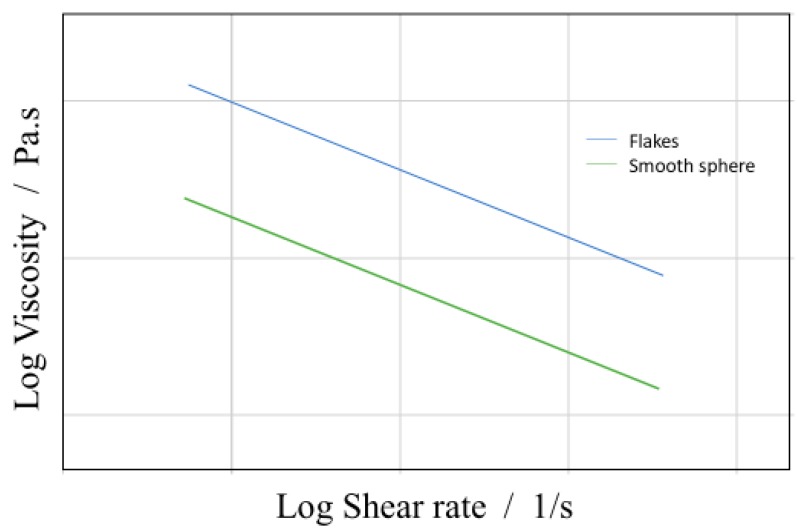
General relationship of viscosity and shear rate, for flake suspension versus smooth sphere suspension (Sketch representation of the general trend concluded from Ran Niu et al. [19]).

The viscosity of the colloidal systems is also dependent on the topography and shape of the suspended particles. From Reinhardt et al., a correlation between the shape of a suspended particle (flakes suspensions as in graphene-based inks) and its viscosity are determined, shown below in Figure 4 [18]. This shows that the shear-thinning behavior in the shear rate *γ* $\overset{\cdot }{<}$ 1 s^−1^ is intensified for flake suspensions, giving similar viscosity within the shear-thinning region, while generally having higher viscosity compared to the sphere-shaped suspensions, as seen in Figure 4. This would likely be associated with the high particle aspect ratio involved with flakes. Particle-particle effects increase due to the orientating of the flakes at higher shear rates, this creates a second Newtonian plateau in the colloid. This specific shear thinning region becomes important when considering manufacturing parameters, as the shear rate is design specific. Although, this applies to the zero-shear rate, this does not describe the effects of the shear rates above the linear viscoelastic region. Steady shear causes de-agglomeration and alignment behaviors of large agglomerates causing strong shear thinning [19].

#### 2.3.5. Surface Tension in Printing

**Figure 5:**
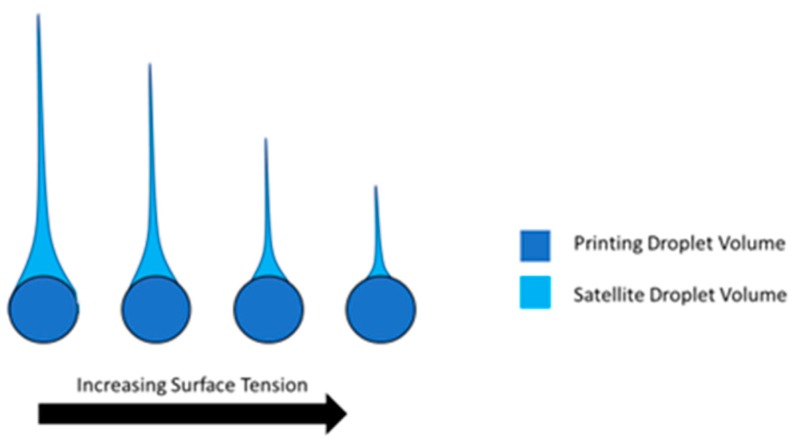
Comparison of droplet volume to satellite volumes with increasing surface tension. As droplets are calculated by their diameter in additive manufacturing, the satellite droplets and tail are not accounted for and cause error. For effective printing, the closer the droplet can realize a spherical shape, the less the error.

Surface tension is an important property for the additive manufacturing printing process, as it can affect the printability and formation on the substrate. Higher surface tension shrinks more rapidly and has a far shorter tail, which leads to fewer satellite droplets. Higher surface tension in the nozzle obtains higher printability and resolution with fewer defects from satellite drops. Leakage and liquid accumulation at the nozzle are also problems associated with low surface tension, or viscosity for that matter [13,14]. The effects of surface tension on ink droplet formation are depicted in Figure 5.

On the other side, high surface tension leads to problems on the substrate, as surface wettability decreases. This can lead to the agglomeration of the printed droplets. The high contact angle formed by the drops due to high surface tension causes the droplets to combine together, either decreasing resolution or causing an error in the build [20]. On the other hand, high wettability increases the image resolution due to lower ink spreading. As concluded by Vafaei et al., as wettability decreases, continuous lines become more difficult to print. It is also noted that the cross-sectional area and volume of printed lines increases with decreasing wettability [20].

The surface tension of the substrate also plays an important role during ink-jet printing. The surface must have higher surface tension than the ink, along with attractive forces to allow for transfer, which in turn gives good adhesion of the print to the substrate. Wettability of the droplets to the surface can determine the feature size (resolution) and cross-section. The values of surface tensions and solubility for various solvents of interest are given in Table 1.

### 2.4. Relation to Inkjet Printing

The importance of characterizing rheological properties is well known in additive manufacturing. Carefully measured rheological parameters are paramount for simulations, which are becoming an integral part of the additive manufacturing process recently. In additive manufacturing, the ink is subjected to shear flow over a wide range of shear rates, which is described through steady-state jetting. The knowledge of rheological properties is essential for process design and optimization. To estimate the value of Force *F* exerted on a fluid, for a real jet discharge from a small orifice, of area A and uniform velocity *V*, the following equation is used:

(9) $$\frac{F}{2\pi \rho v^{2}}\approx \frac{1}{2\pi }\frac{AV^{2}}{v^{2}}$$

where *ρ* and *v* are the density and dimensional consistency constant. The mass flux through this orifice too can be defined:

(10) $$\rho AV=\frac{F}{V}$$

The dimensionless parameter $\frac{F}{2\pi \rho v^{2}}$ in Equation (9) is known as the Reynolds number, this is generally depicted as:

(11) $$Re=\frac{\rho VL}{\eta }$$

where *V*, *L*, and *η* is the velocity of the fluid with respect to the object (m/s), characteristic linear dimension (m) and the dynamic viscosity of the fluid (Pa·s), respectively [22]. Low values for the Reynolds number signify high viscosity, high values signify low viscosity, which usually leads to satellite droplet formation. For an idealized printability though, this number alone does not tell the whole story. 

Another dimensionless value of interest is the Weber number, which is the characteristic number describing droplet formation ability. Two forces form the basis of the Weber number, one being the fluid-mechanical force and the other being surface tension. When a liquid flows through a second fluid phase, either a gas or a liquid, then the fluid-mechanical force *F_A_* causes the drops to deform and ultimately disperse:

(12) $$F_{A}=\frac{1}{2}C_{w}\frac{\pi }{4}L^{2}\rho v^{2}$$

*C_w_*, *L*, *ρ* and *v* is the Drag coefficient, Characteristics length, Density and Flow rate respectively. Surface tension involves a cohesion force *F_k_*, opposes the increase in surface area, which is caused by the falling deformation. The droplet is held together by:

(13) $$F_{k}=\pi L\sigma$$

The Weber number is the ratio of these forces and hold the following relation: [22]

(14) $$W_{e}=\frac{8F_{A}}{C_{w}F_{k}}=\frac{\rho v^{2}L}{\sigma }$$

**Figure 6:**
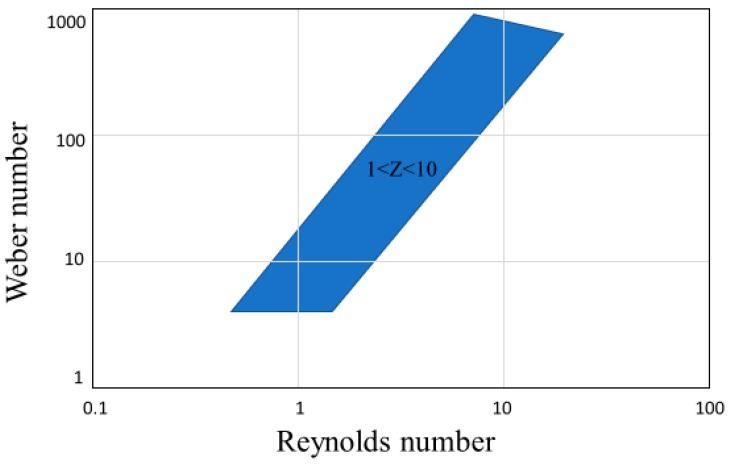
Graphical representation of the idealized region for stable printing, where 1 < *Z* < 10, the inverse of the Ohnesorge number. The depicted blue area is the Goldilocks zone for 3D printing.

A combination of Reynolds and Weber numbers provide an understanding of fluid drop formation, the Ohnesorge number, defined as:

(15) $$Oh=\frac{\sqrt{We}}{Re}=\frac{\eta }{\sqrt{\rho L\sigma }}$$

where *η* and *σ* are the dynamic viscosity and surface tension of the fluid, respectively [22]. This value is normally utilized as its inverse *Z* = 1/*Oh*, where 1 < *Z* < 10 are the limits to stable drop formation [23]. This idealized region for printability is seen in Figure 6.

These equations describe few of the defining properties of printability for an ink like dynamic viscosity, density, characteristic linear dimension (Nozzle diameter), and surface tension. The accuracy of the calculations from the above equations is dependent on the printing parameters such as the waveform and temperature, which also have an effect on the printability [24]. The values are not ubiquitous, Lee et al. have shown nozzle clogging causing nonjetting even in viable *Z* values. The observed printability at a *Z* range of 2.5 < *Z* < 26 for Newtonian fluids, however, they were unable to jet colloidal ZnO suspensions for the identical range [4]. 

The Carbon-based colloidal suspensions present issues in terms of printability and their rheological properties. The largest problem associated with printing carbon-based inks is clogging of the nozzle and print head. Clogging occurs as a result of the suspended carbon particles agglomerating, preventing an even flow through the print head. Special care must be taken for designing carbon-based inks, to ensure consistent flow. Another rheological issue is satellite or unintentional trailing droplets, arising from low viscosity, which compromises the print accuracy and precision. The additions of surfactants in the ink to improve particle distribution further exasperate the issue since they lead to larger satellite drops [25]. Viscosity and density ratios also play a major role in droplet formation. Low-density ratio leads to larger satellite droplet formations, similarly, lower ink viscosity has been reported to give way for easier satellite droplet formation [26]. On the other hand, low viscosity is a necessity for printability, therefore compromises have to be made, and all important parameters like conductivity, mechanical strength, flexibility, particle size, flow properties must be carefully optimized (as shown in Figure 2) during the ink design process to suit a specific printer. Speed and precision of print rely heavily on the ink viscosity, with pinching speed of droplets being proportional to surface tension and inversely proportional to viscosity [27]. Typical viscosities for ink-jet printing are between 5 and 20 mPa·s and surface tensions of 25 to 35 mN/m. Though much like Z-numbers, this is not a ubiquitous rule for 3D printing [28]. The zero shear viscosity of various printable carbon inks is shown in Table 2.

At a given shear rate through the nozzle of radius (*R*) and length *L*, the pressure required to extrude a shear thinning liquid, such as carbon-based suspensions, can be calculated by:

(16) $$\Delta p=\frac{8\eta QL}{\pi R^{4}}$$

where *Q* is the volumetric flow rate and *η* is the viscosity [37]. This equation presents the importance of the radius and length of the nozzle in ink design. The radius of the nozzle is a major factor for printing resolution and droplet formation. 

Methods for producing droplets from the printing nozzle can be split into three methods. One is continuous inkjet printing, where a continuous ink stream is broken into droplets of uniform size and spacing [38]. The nozzle is held at a potential relative to ground that transfers charge to the drops. Deflector plates are utilized to steer droplets, as due to the continuous flow of drops, unwanted drops are deflected into a gutter to be recycled back.

Another method of producing droplets is drop-on-demand technologies, allowing the printhead to produce singular droplets. This process is normally driven by the application of voltage pulses to a piezoelectric actuator, creating pressure through its mechanical motions. Optimization of voltage is required dependent on material, nozzle dimensions and environment [39]. Ejected columns of liquid are pinched off to form a drop. The volume ranges are from 1 pL–1 nL, with a diameter range of 10–100 μm [40].

Modeling plays a role in optimizing these nozzles, with issues such as clogging. Computer-based simulation allows for rapid experimentation and parameter variation in an aim to optimize and predict the most effective system. Barati et al. [41] presented a model for reducing clogging through transient simulation. Looking at the wall-fluid adhesion mechanisms and interactions, clogging could be simulated, and nozzle design can be varied so as to reduce clogging. Simulations are then followed up by a validation experiment, verifying the simulations results [42].

## 3. Carbon Based Inks

### 3.1. Carbon Based Inks—A Colloidal Suspension

Carbon conventionally is a hard solid in nature, which stems from the covalent bonding between carbon atoms. Direct printing of carbon alone would be impossible, with the exception of extreme combinations of temperature and pressure. Hence, to utilize carbon forms in additive manufacturing, the particles must be assisted by a liquid, acting as a vehicle to make printing possible. This combination of solid particles in a liquid is known as a colloidal system.

#### 3.1.1. Colloidal Systems

A colloid is a heterogeneous solution, with a dispersed phase uniformly distributed throughout the second medium, the dispersion phase. When the dispersed phase is smaller than 1nm in diameter, the system assumes the properties of a true solution. Conversely, when this dispersion is larger than 1000 nm, the separation is large enough that it is considered a suspension. Suspensions containing much larger solid particles or high solid content can form sedimentation [43].

#### 3.1.2. Einstein Viscosity & the Krieger–Dougherty Equation

From the energy dissipation calculation of suspensions, Einstein derived that a dilute suspension of rigid spherical particles behaves as a Newtonian fluid with relative viscosity (*η_r_*) represented by the following equation:

(17) $$\eta_{r}=1+2.5\Phi$$

where Φ is the volume fraction of the dispersed phase. This assumes that all particles are separated by such a distance so that there is no interaction between them.

(18) $$\Phi =n4\pi \frac{a^{3}}{3}$$

where *n* is the number density of particles and a is the particle radius [44]. When looking for higher particle concentrations, Krieger and Dougherty proposed a semi-empirical equation for the concentration dependence of the viscosity:

(19) $$\eta_{r}={\left(1-\frac{\Phi }{\Phi_{max}}\right)}^{-2.5\Phi_{max}}$$

where $\Phi_{max}$ is the maximum packing fraction or the volume fraction at which the zero shear viscosity diverges. When particle packing density is low, this reduces to the Einstein relation. Approaching the maximum volume fraction $\Phi_{max}$ the particle packing density is such that the dispersion flow is impossible and $\eta_{r}\to \infty$ [45]. This increase in viscosity is attributed with the increase in particle concentration in a suspension. The restriction in the relative motion of the particles results in an increase of the particle collision in the suspension, subsequently leading to an increase in the frictional forces. The theoretical models explaining colloidal systems assume hard sphere systems that only interact through hydrodynamics, where the distribution of particles is highly sensitive to the shape, size, and surface charge of the particles in suspension [44]. However, real colloidal suspensions lack the hard sphere shape, which further affects aggregation and flocculation at higher concentrations of particles and significantly alters the system’s flow properties. This promotes non-Newtonian rheological behavior.

Adding particles in the ink does not simply increase the viscosity of the liquid as a result of the hydrodynamic disturbance of the flow, it can also be a cause for a deviation from the Newtonian behavior, including shear rate dependent viscosity, elasticity, and time-dependent rheological behavior. Colloidal dispersions at low to moderate volume fraction exhibit shear-thinning behavior analogous to low viscous liquids. On the other hand at high concentrations they behave like solids, which require higher stress to start the flow. The rheological behavior of a suspension is strongly dependent on the nature of colloidal interactions attraction. Depending on whether the colloidal interactions are attractive or repulsive, the particles can form different structures, which determine the rheological behavior of the material [46].

### 3.2. Graphene

Graphene is a transparent two-dimensional sheet of carbon atoms arranged in hexagons. Graphene is the single layer equivalent of graphite. Much interest and research have been conducted on graphene due to its exceptional electrical and mechanical properties. Because of these properties, there is a growing interest in additive manufacturing and printing, opening possibilities to light, strong, and conductive builds.

Jakus et al. [47] demonstrated 3D printed biocompatible scaffolds from graphene inks, which show in vivo compatibility over at least 30 days. This opens up biomedical usages of graphene printed inks, which can be applied to in vitro and in vivo tissue regenerative engineering applications. The potential biocompatibility of Graphene opens completely new realms of applicability for graphene due to its combination of conductivity and printability. Though, there are other reports in the literature that conflict on this biocompatibility, with factors such as surface functionalization (which reduces toxicity and is in most cases a must for functional use), size and shape all playing effective roles in possible toxicity. [48] Zhang et al. and Shen et al. [22,23] discussed the potential for graphene-based inks in drug deliverance, gene therapy, cancer therapy, tissue engineering, biosensing, and bioimaging. Recently, Graphene 3D Labs have produced conductive graphene composite filaments for Fused Filament Fabrication in the commercial market. 

### 3.3. Graphene Oxide

Graphene oxide is a functionalized Graphene, created through the oxidation of graphite. The oxidation expands layer separation in the graphite and makes the layers hydrophilic, allowing for dispersion in water. Sonication exfoliates the graphite further, creating single and few-layer GO. Importantly to the functional use of GO, the lower the oxygen content, the more conductive. GO forms non-covalent networks with optimum rheological properties with respect to printing. Shear thinning behavior of the colloidal suspensions along with the relatively high storage modulus (*G’*) gives strong printability and self-supporting structures. Reduced graphene oxide (rGO) is the restoration of pristine graphene properties to GO through the reduction, removal of Oxide. This is of value due to the ease at which GO can be handled and suspended in water, but the need to recuperate conductivity properties in the end product [29,30].

Due to the highly anisotropic nature of graphene sheets, with the thickness of the sheet being in single atomic layers and the lateral being in the micrometers scale, the properties of the graphene oxide are dependent on how it is assembled. Careful control of the assembly of the flakes then is of necessity, as agglomerations, bends and crimples will affect the properties of the final product. For additive manufacturing, the directional dispersion of the flakes after printing is crucial to the effective properties. Kim et al. [49] studied the surface activity of GO in a suspension and have shown that graphene aligns with gas bubbles. GO inks have also been used in electronics, with lithium-ion battery electrodes fabricated through the use of high viscosity GO-based electrode inks by Fu et al. [50]. 

Using 2D and 3D graphene printed inks, both planar and volumetric structures can potentially be made with this material. Graphene retains mechanical flexibility, high electrical conductivity, and stability to thermal and chemical effects after deposition [51]. Due to Graphene’s mechanical properties, its use in composites for printing mechanical reinforcement is well documented [2,30,31]. 

Interest is also around the flexible nature of graphene prints. The combination of conductivity and its flexible nature open new paradigms of consumer electronic capabilities. This conductive and flexible nature was investigated by Secor et al. for inkjet printing. They developed a graphene/EC powder that was produced at room temperature and was capable of stable jetting of features, boasting excellent printability and geometrical shaping [52]. On the toxicity of Graphene and Graphene Oxide, the available data is still insufficient for conclusive answers [53].

Table 2 presents the conductivity of reduced graphene oxide-based inks, from Fernandez-Merino et al. [54].

The higher concentration of rGO is key to higher conductivity. The conductivity of 100% rGO far exceeds the conductivity at lower concentrations, rGO/SDS closest, with similarly high rGO concentrations in wt%. Uddin et al. [55], studied the impact of surfactant on conductivity, as shown in Table 3. Covalent dispersion techniques also are investigated, from work by Kuila et al. [56] in Table 4.

### 3.4. Carbon Nanotubes

Carbon nanotubes (CNT) are similar to Graphene with great interest and research being conducted due to its extraordinary, electrical, optoelectronic, and biosensing capabilities [25,26,27]. There is a wealth of literature on the mechanical, electrical, and thermal properties of carbon nanotubes [45,46]. Owing to these properties, a whole plethora of applications where carbon nanotubes could be used. Additive CNTs are often combined with the polymers to form strong, electrically conductive composites [5] or in water suspensions for nanoelectronics and sensors [57].

Inkjet printing of carbon nanotubes has been demonstrated, notably by Kordas et al., to create conductive patterns, [58] using carboxylated Multi-Wall Carbon Nanotubes (MWCNTs). The notable advantage of CNTs over other conventional conductive inks is the lack of carbon nanotubes to require curing. Electrically conductive CNT-based inks have been designed for dip coating and screen-printing methods by Shin et al. [59]. These samples proved to be highly flexible, bendable, and stretchable while maintaining electrical connectivity and very little change in resistance. Single-Walled Carbon Nanotubes (SWCNTs) have been printed with inkjet printers as a thin film, the flexible electrode on cloth by Chen et al. [60]. Control over geometry and pattern, in this case, showed promise for wearable energy storage, as a printable electrochemical capacitor.

### 3.5. Carbon Black

Carbon black is a finely particulate paracrystalline carbon produced by the incomplete combustion of heavy petroleum products or vegetable oil. [61] Carbon black has been used as part of compounds due to its additional mechanical strength, conductivity, black pigmentation, and absorption of ultraviolet light [44,45]. Talarico et al. have designed carbon black based electrochemical sensors by using a screen printing process. Printing energy storage devices and supercapacitors have also been fabricated from carbon black inks [46,47]. Inkjet printed carbon black composites have also been used as a catalyst layer in fuel cells due to their high conductivity and corrosion resistance, presented by Taylor et al. [62]. One of the advantages of carbon black is that it has a history of being used in lithographic inks and black inkjet printers, the printing process is well established. 

### 3.6. Carbon Fiber

Carbon fiber is a well-established and go-to engineering material due to its high strength mechanical properties and light-weight. Using carbon fibers in additive manufacturing is still less established though. However, the strength of carbon fiber comes from the length of the fibers, however, the additive manufacturing process requires a small enough length of the fibers, however, the additive manufacturing process requires a small enough length to fit through the nozzle of the printer; therefore, compromises have to be made on the printing speed. 

Tekinalp et al. achieved highly orientated carbon fiber-polymers (0.2–0.4 mm) through Fused Filament Fabrication (FFF). Additive manufacturing here controls the orientation and allows for good dispersions, but also presents higher porosity, which is detrimental to the improvement seen in the orientation [63].

Because of the relation of carbon fibers strength with length and orientation, printing methods with continuous carbon fibers have been investigated. A methodology for in-nozzle impregnation has been demonstrated by Matsuzaki et al. wherein, the carbon fiber is fed through the nozzle with polylactic acid (PLA) in one continuous line [64]. Through the printing of carbon fiber with PLA, Tian et al. tested and optimized conditions necessary for continuous fiber printing [65]. Table 5 summarizes the relationship of conductivity with print thickness for various type of ink compositions.

Looking at all samples, a comparison of the methodology of a few ink compositions was made, observing the conductivity found in Table 5.

## 4. Problems Associated with Printing Carbon Based Inks

### 4.1. Agglomeration

One of the major concerns for carbon-based inks is its reaction with the liquid medium in which it is suspended. Due to the hydrophilic nature of aromatic carbon forms, water, the first choice for ink suspension, however, water-based inks suffer agglomeration. Water-based inks are ideally suited due to their environmentally friendly nature, the ease at which they can be stored and handled [66]. Agglomeration is a challenge both for additive manufacturing due to limitations of the nozzle area and in graphene, the restacking of sheets to form graphite, which possesses inferior properties [67]. One method for avoiding the hydrophilic nature of graphene is through functionalization of the graphene sheets, an example of such is graphene oxide, as discussed earlier on [26,68]. The oxidation of graphene results in a reduction in the conductivity, which is undesirable.

Similarly, for CNTs, three methods are taken to counter this problem. One is the functionalization of the side walls of the CNT. This is generally done with Carboxylation, the addition of hydrophilic carboxyl (−COOH) groups to the carbon nanotube walls. Carboxylation can prove to be counterproductive as it decreases the conductivity and hence, effectiveness [58].

Sonication is another method commonly used for CNTs and graphene-based inks. Sonification is the irradiation of a liquid sample with ultrasonic (>20 kHz) waves. These high-frequency sound waves propagate in the liquid, resulting in high-pressure and low-pressure cycles, creating agitation in the medium [69]. Another common solution to avoid agglomeration is the addition of dispersants in the solvent to avoid agglomeration. The use of polymers and surfactants have been utilized to this end, by coating the CNTs, Van der Waal forces can be suppressed [70]. The presence of both hydrophilic heads and hydrophobic tails in the dispersants are known to disperse the CNTs and colloids in the inks with a huge reduction in agglomeration [71].

On the other hand, the problem of hydrophobicity altogether by utilizing organic solvents in the inks in place of water. Organic solvents can avoid the conglomeration of the CNTs effectively without functionalization and there is no compromise on the conductivity. The organic solvent molecules are attracted to the surface of the CNT due to its hydrophobic nature, which prevents the Van der Waal attraction of the CNTs [63,64].

Though organic solvents present their own set of problems, as they pose a hazard to the environment and health. Careful cartridge design and disposal are of importance since most of the organic solvents can potentially be highly corrosive. These hazards need to be properly addressed, especially when the potential use is in medical applications. Most of the organic solvents are highly volatile and evaporate faster, despite lower surface tension compared to water. The evaporation rate of the solvents needs to be properly optimized according to the printing method, otherwise, the ink may clog the nozzle and agglomeration could result through a loss of solvent [65,72].

### 4.2. Maintaining Suspension and Dispersion

The initial aggregation rate for GO flakes can be described as:

(20) $$k_{a}N_{0}\propto {\left(\frac{dR_{h}\left(t\right)}{dt}\right)}_{t\to 0}$$

where *N*_0_ is the initial particle concentration, *R_h_* hydrodynamic radius. From this the aggregation attachment efficiency *α* (quantification of particle aggregation kinetics)

(21) $$\alpha =\frac{1}{W}=\frac{k_{a}}{k_{a,fast}}=\frac{\frac{1}{N_{0}}{\left(\frac{dR_{h}\left(t\right)}{dt}\right)}_{t\to 0}}{\frac{1}{{\left(N_{0}\right)}_{fast}}{\left(\frac{dR_{h}\left(t\right)}{dt}\right)}_{t\to 0,fast}}$$

“*fast*” here refers to favorable aggregation conditions [73].

The shelf life of a 3D ink is also important for practical use in industry. The main concern with carbon-based suspensions is the stability, the settling, and agglomeration of particles with time. Methods such as solvents that maintain constant dispersion [74] dispersing using sonication [71] or the addition of copolymers to increase stability [75]. Su et al. conducted testing on colloidal stabilities of high concentration graphene inks, 1 mg/L–3 mg/L suspensions have a constant distribution for at least as an hour, whereas concentrations of graphene above 3 mg/L only have a shelf life of less than one minute [76]. An environmental impact study into GO in water by Chowdhury et al. investigated the stability of GO nanoparticles in various water types, 10 mg/L was shown to be stable in fresh water for almost a month [77]. Comparisons of different dispersion methods for carbon-based inks are shown in Table 6.

### 4.3. Health, Safety, and Environmental Concerns

As mentioned previously, the biocompatibility of carbon nanoforms still requires further investigation. A review into the potential insurability of such nanoparticles has been carried out by Mullins et al., in which the minimization of exposure and framework for the transfer of technology is established [85]. The extent or potential of harm from these nanoforms could potentially damage the use of graphene and CNTs in personal electrical and medical devices. Graphene presents a number of potential issues ranging from environmental risks and toxicity, due to the nanoscale, which also reveals the difficulties related to removing and filtering the particles [86]. CNTs can also cause damage due to their scale, with oxidation stress and biocompatibility. Factors that appear to affect this are length, diameter, purity, production method, and functionalization, and that by modifying these factors, CNTs may be safe for human use [87]. The majority of the solvents that are utilized in printable inks technology present environmental health risks, [88] from handling to evaporation, hazards pertain. 

Though, accounting for well-established inks and historical influence, printing is an environmentally damaging operation, especially when considering the heavy metals and volatile organic solvents involved [80,81]. Comparing the other conductive inks utilized in additive manufacturing, silver nanoparticles similarly contain potential hazards to the environment [89]. The relative unknown level of environmental risk from Carbon nanoforms is comparatively lower than the established heavy metal and organic solvent hazards [90].

## 5. Applications of Printable Carbon Inks

### 5.1. Electronics

Digital circuits have been printed using CNT at sub-3V voltages by Ha et al. onto plastic substrates [91]. Nanowires have been printed as nano-arches using rGO suspended in water by Kim et al. [92]. These nanowires were functionalized in a gas sensor prototype as a 3D transducer. 

#### 5.1.1. Transistors

Printed Graphene thin film transistors have been demonstrated to have electron mobility up to ~95 cm^2^V^−1^s^−1^ by Torrisi et al. [79]. The fabrication of field effect transistors through inkjet printing of graphene has numerous examples. [93,94,95] Carbon nanotubes are also presenting very promising results as thin film transistor, exhibiting properties similar to CMOS devices [93,94]. Showing the potential viability of flexible, transparent electronics, created from additive manufacturing carbon based inks. Paper printable transistors of carbon black and rGO have been developed, presenting the flexibility of carbon transistors developed through additive manufacturing [96]. With graphene being suggested as the long term air to silicon in conventional computing, [97] additive manufactured transistors will allow for rapid testing and design.

#### 5.1.2. Sensors

Sensors have been designed using polymer/carbon black composites, by Loffredo et al. [98]. Highly stretchable sensors based on embedded based on embedded 3D printing of carbon-based resistive ink within an elastomer [99]. Graphene in this ink is used to add conductivity along with its elastic properties to retain the desired elastomers use. GO and FGO based inks have been shown to be designable for sensors directly with standard office inkjet printers while maintaining high electrical conductivity [78]. Glucose biosensors are one such example of carbon-based ink demonstrating the practical electrical properties of GOs from inkjet printing [100].

#### 5.1.3. Electrodes

The first work into GO-based electrode inks for use in lithium-ion battery prototypes using 3D printing has been designed by Fu et al. with optimization of the viscosity and viscoelastic properties [50]. This 3D printed electrode exhibited stable cyclic performance with an LTO anode, with specific capacities of ≈160 mAhg^−1^ (LFP) and ≈170 mAg^−1^ (LTO). Electrodes made from 3D printed graphene/PLA were demonstrated by Browne et al., these were electrochemically treated for higher conductivity [101]. Carbon nanotube inks present numerous examples of electrode capabilities [102,103,104].

#### 5.1.4. Supercapacitor

Yao et al. in 2018 printed a record-breaking capacitance with a graphene-based scaffold and pseudocapacitive electrodes of Manganese Oxide (MnO2). This shows promise for the feasibility of practical pseudocapacitive electrodes [105]. This was further improved upon with the “wrapping” of the supercapacitor with CNTs [106]. GO has also been utilized in the design of All-Solid-State, flexible Micro-supercapacitors. Pei et al. [107] demonstrated this using a carbon-based hybrid ink using GO, showing promising potential for lightweight energy storage. A novelty of additive manufacturing allows for full packaging of electrical components during the printing process, supercapacitors of this elk have been designed by Chen et al. for SW-CNTs [108].

### 5.2. Biological Scaffolding

Lee et al. utilized Multi-Wall CNTs (MWCNT) with PEGDA polymer to print an electroconductive scaffold for nerve regeneration through therapeutic electrical stimulation [109]. Similar lines to this, Ho et al. fabricated a composite scaffold using CNT and polycaprolactone (PCL) with biological compatibilities to cardiac tissue engineering using a CNT based 3D ink [110]. Bone cell growth has been presented using PCL-hydroxyapatite scaffolds filled with CNTs, to stimulate cell growth [111]. Graphene too can be utilized for cell regeneration, with Jakus et al. showing the possible use of graphene-based inks in biological scaffolding [7]. The carbon forms are utilized in each of these composites act to add conductivity and protein absorption to the polymers, promoting faster cell growth. Conductivity is important to stimulate cells with electrical pulses. A summary of various applications of carbon inks suggested in the literature are shown in Table 7.

Summating the applications of carbon based inks is seen in Table 8 below.

## 6. Conclusions

Since its inception in the 1980s, additive manufacturing has become a technology of choice due to its ability for the rapid prototyping (RP) of complex shapes and geometry directly from Computer Aided Design (CAD). Despite huge interest, the technology still suffers some technological barriers that hinder its use in wider applications. Main areas of concern are quality of materials (inks), limitations of equipment, optimization of the manufacturing process and lack of self-correction during the printing process. These limitations needed to be addressed through improvements in the instrument design and optimization of the process.

In this paper, state of the art additive manufacturing of carbon based materials is described. Carbon nanoforms possess huge potential for industrial application through the creation of superior properties in advanced composites. One of the key properties of carbon nanoforms that makes them suitable for many applications is that they offer a range in conductivities of the printed materials, however, it is strongly size dependent. The particle size on the other hand controls the ink rheology and printability. Therefore, due to the nature of the composites, printing process compromises are made to the properties of these idealized carbon forms to make them printable. The paper also discusses the reliance of printability on the rheological and flow properties of the ink. The rheological characterization and understanding of the flow behavior at various shear rates and material loadings helps to assess ink processability and optimization of the process design. Another reason for understanding material rheology is to simulate and link the flow behavior with the actual printing process which is becoming an integral part of the additive manufacturing process. The paper highlights several issues encountered by graphene and carbon nanotube-based materials, one of the main problems being their poor solubility in water, which leads to problems in terms of rheology and dispersion. While the oxidation of these nanoforms improves it, oxidation has a negative effect on conductivity, a pivotal property of the material for many applications. With considerate design of the material and slurry, carbon based ink can be optimized to produce inks with the desired properties. The combination of high quality inks with versatile design capabilities and additive manufacturing could revolutionize the consumer, medical, and industrial electronics.

## Figures and Tables

**Table 1:** Surface tensions and solubility parameters of solvents for GO, reduced graphene oxide (rGO). Adapted from Konios et al. [21].

Solvent	Surface Tension (mN/m)	GO Solubility (μg/mL)	rGO Solubility (μg/mL)
De-ionized water	72.8	6.6	4.74
Acetone	25.2	0.8	0.9
Methanol	22.7	0.16	0.52
Ethanol	22.1	0.25	0.91
2-propanol	21.66	1.82	1.2
Ethylene glycol	47.7	5.5	4.9
Tetrahydrofuran (THF)	26.4	2.15	1.44
N,N-dimethyformamide (DMF)	37.1	1.96	1.73
N-methyl-2-pyrrolidone (NMP)	40.1	8.7	9.4
n-Hexane	18.43	0.1	0.61
Dichloromethane (DCM)	26.5	0.21	1.16
Chloroform	27.5	1.3	4.6
Toluene	28.4	1.57	4.14
Chlorobenzene (CB)	33.6	1.62	3.4
o-Dichlorobenzene (o-DCB)	36.7	1.91	8.94
1-Chloronaphthalene (CN)	41.8	1.8	8.1
Acetylaceton	31.2	1.5	1.02
Diethyl ether	17	0.72	0.4

**Table 2:** Examples from the literature of 3D printable carbon nanoform suspensions (zero shear viscosity).

Carbon Form	Ink Method	Zero Shear Viscosity/Pa·s	Reference
Graphene	Organic solvent	~18.5	[29]
	Organic solvent + dispersant	~6	-
	Water 0.5%	0.478 mPa·s	[30]
	Water 1%	~ 0.52 mPa·s	-
	Water 1.5%	~ 0.56 mPa·s	-
Graphene Oxide	LC viscoelastic gel 2 mg/mL	~3.8	[31]
	LC viscoelastic gel 9 mg/mL	~100	-
	PMMA matrix 0.05% GO	~80	[32]
	PMMA matrix 1.2% GO	~20,000	-
Carbon Nanotubes	epoxy resin 0.3% treated CNT	20	[33]
	PIB 1.7% MWCNT	~1	[34]
	PIB 3.0% MWCNT	~7	-
	PIB 6.0% MWCNT	~900	-
	PDMS 1% MWCNT	~10	[35]
	PDMS 4% MWCNT	30–40	-
Carbon Black	Poly-acrylate, 5.3% spherical	~0.6	[36]
	Poly-acrylate, 11% spherical	~6000	-

**Table 3:** This is a table presenting the Conductivity properties of various tested reduced graphene oxide-based inks. Reproduced with permission from M.J.Fernández-Merino et al., Carbon; published by Elsevier, 2012 [54].

Film	rGO (wt%)	Conductivity (S·m^−1^)	Specific Capacitance (F·g^−1^)
rGO	100	7548	38
rGO/PBA	36	13.31	1
rGO/DOC	47	0.06	1
rGO/TDOC	36	2.18	3
rGO/PSS	41	10.51	114
rGO/SDBS	29	0.87	7
rGO/SDS	87	4679	46
rGO/CHAPS	36	0.92	2
rGO/DBDM	11	0.01	3
rGO/P-123	38	5.53	12
rGO/Brij 700	10	1.08	6
rGO/Tween 80	13	0.41	95

**Table 4:** Comparison of materials of absorbed surfactant and electrical conductivity. Reproduced with permission from Md. Elias Uddin et al., Journal of Alloys and Compounds; published by Elsevier, 2013 [55].

Sample	Adsorbed Surfactant (%)	Conductivity (S·m^−1^)
GO	34.34	0.002
CR-G	-	4760
SDBS-0.25-G	7.02	108
SDBS-0.5-G	6.13	106
SDBS-1-G	9.31	97
SDS-0.25-G	17.41	94
SDBS-0.5-G	17.93	93
SDBS-1-G	21.62	95
TRX-0.25-G	9.63	98
TRX-0.5-G	9.63	92
TRX-1-G	9.37	89

**Table 5:** Comparison of covalent dispersion techniques dispersibility and electrical conductivity. Reproduced with permission from Tapas Kuila et al., Progress in Materials Science; published by Elsevier, 2012 [56].

Modification Techniques	Modifying Agent	Dispersing Medium	Dispersibility (mg/mL)	Electrical Conductivity (S·m^−1^)
Nucleophilic Substitution	Alkyl amine/amino acid	CHCl_3_, THF, toluene, DCM	-	-
	4-Aminobenzene sulfonic acid	Water	0.2	-
	4,4’-Diaminodiphenyl ether	Xylene, methanol	0.1	-
	POA	THF	0.2	-
	Allylamine	Water, DMF	1.55	-
	APTS	Water, ethanol, DMF, DMSO	0.5	-
	IL-NH_2_	Water, DMF, DMSO	0.5	-
	PLL	Water	0.5	-
	Dopamine	Water	0.05	-
	Polyglycerol	Water	3	-
	Poly(norepinephrine)	Water, methanol, acetone, DMF, NMP, THF	0.1	-
Electrophilic Substitution	ANS	Water	3	145
	4-Bromo aniline	DMF	0.02	-
	Sulfanilic acid	Water	2	1250
	NMP	Ethanol, DMF, NMP, PC, THF	0.2–1.4	21,600
Condensation Reaction	Organic isocyanate	DMF, NMP, DMSO, HMPA	1 (DMF)	-
	Organic diisocyanate	DMF	-	1.9 × 10^4^
	ODA	THF, CCl_4_, 1,2-dichloroethane	0.5 (THF)	-
	TMEDA	THF	0.2	-
	PEG-NH_2_	Water	1	-
	CS	Water	2	
	TPAPAM	THF	-	-
	β-CD	Water, acetone, DMF	1 (DMF)	-
	α-CD, β-CD, γ-CD	Water, ethanol, DMF, DMSO	>2.5	-
	PVA	Water, DMSO	-	-
	TPP-NH_2_	DMF	-	-
	Adenine, cystine, nicotamide, OVA	Water	0.1	-
Addition Reaction	POA	THF	0.2	-
	Polyacetylene	Ortho dichlorobenzene (O-DCB)	0.1	-
	Aryne	DMF, O-DCB	0.4	-
	Cyclopropanated malonate	Toluene, O-DCB, DMF, DCM	0.5	-

**Table 6:** Various carbon based ink examples from the literature, showing the relationship of ink medium with conductivity and film.

Carbon Form	Ink	Conductivity (S·m^−1^)	Thickness of Prints	Reference
Graphene	Pristine	~40,000	-	[72]
	GO + water	~400	20 prints	[78]
	Few layer GO + water	~875	20 prints	[78]
	G + NMP (Substrate O_2_ plasma treated)	~0.08	50 nm	[79]
	G + NMP (Substrate Pristine)	~30	50 nm	[79]
	G + NMP (Substrated HMDS-coated)	~95	50 nm	[79]
	G + Cyrene	37,000	7.8 μm	[80]
Carbon Nanotube	SWNT + water + SDBS (substrate paper)	~550	50 nm	[81]
	MWCNT 12% + PAN + DMF	~100	300 nm	[82]
	MWCNT 89% + PAN + DMF	~333	300 nm	[82]
	MWCNT + aqueous solution	2400 ± 180	10 μm	[59]
Carbon Black	Cold microwave plasma, CO_2_ 1.7%	256	-	[83]
Silver	Ag microparticles + Organic binder + solvent (Substrate PET/glass)	46,700	Screen printed	[84]

**Table 7:** A table presenting the advantages and disadvantages of methods of dispersing carbon-based materials, adapted from Liang et al. under open access © School of Materials Science and Engineering, Southwest Jiaotong University, Chengdu 610031, China [112].

Dispersion Method	Mechanism	Advantage	Disadvantage
Physical methods	Applying physical force to separate agglomerated graphene	Simple operation	Low dispersion rate and possible damage to nanoparticles
Covalent bonding methods	Introducing various active groups by chemical reaction on the surface or edge of the graphene	Making the graphene more workable and operable	Causing damage to the initial structure of the graphene
Noncovalent bonding methods	Modifying the graphene’s structure with functionalized molecules through non-covalent interaction	Functionalizing carbon forms, allowing ease of use	Introduces other components and impurities to the carbon forms

**Table 8:** Summary table for possible applications for carbon-based inks.

Carbon Form	Ink Method	Application	Reference
Graphene	rGO + water	Nanowire arches	[92]
	Graphene/h-BN + NMP + ethanol	Transistor	[93]
	N-Methylpyrrolidone	Transistor	[79]
	PBT/Graphene composite	Conductive polymer	[113]
	GO + water	Lithium ion battery electrodes	[50]
	Graphene/PLA	Electrodes	[101]
	Graphene/PLA	Energy storage	[114]
	Graphene + Hypromellose, aerogel suspension	Pseudocapacitive Electrodes	[105]
	Graphene + poly-lactide-co-glycolide	Electrical and biomedical scaffolding	-
Carbon Nanotubes	PEDOT:PSS	Digital circuit	[91]
	PBT/CNT composite	Conductive polymer	[113]
	Amine functionalization MWCNT/PEGDA matrix	Nerve regeneration scaffolding	[109]
	CNT + PCL in chloroform	Cardiac tissue scaffolding	[110]
	PCL-hydroxyapatite scaffold + CNT	Stimulate bone cell growth	[111]
Carbon Black	Polymer-carbon black	Chemical sensor	[99]
	Conductive carbon grease (Dimethylpolysiloxane)	Strain sensor	[99]
Carbon Fibers	Active carbon + water	supercapacitor	[115]
	Epoxy	Lightweight cellular composites, controlled alignment	[116]
